# Towards Rapid and Low-Cost Stroke Detection Using SERS and Machine Learning

**DOI:** 10.3390/bios15030136

**Published:** 2025-02-22

**Authors:** Cristina Freitas, João Eleutério, Gabriela Soares, Maria Enea, Daniela Nunes, Elvira Fortunato, Rodrigo Martins, Hugo Águas, Eulália Pereira, Helena L. A. Vieira, Lúcio Studer Ferreira, Ricardo Franco

**Affiliations:** 1 Associate Laboratory i4HB—Institute for Health and Bioeconomy, Faculdade de Ciências e Tecnologia, Universidade NOVA de Lisboa, 2819-516 Caparica, Portugal; cmt.freitas@campus.fct.unl.pt (C.F.); hl.vieira@fct.unl.pt (H.L.A.V.); 2 UCIBIO—Applied Molecular Biosciences Unit, Departamento de Química, Faculdade de Ciências e Tecnologia, Universidade NOVA de Lisboa, 2819-516 Caparica, Portugal; 3 COPELABS—Departamento de Engenharia Informática e Sistemas de Informação, Universidade Lusófona, Centro Universitário de Lisboa, 1749-024 Lisboa, Portugal; a22308295@alunos.ulht.pt (J.E.); p7333@ulusofona.pt (G.S.); 4 LAQV/REQUIMTE—Laboratório Associado para a Química Verde/Rede de Química e Tecnologia, Departamento de Química e Bioquímica, Faculdade de Ciências, Universidade do Porto, 4169-007 Porto, Portugal; menea@fc.up.pt (M.E.); eulalia.pereira@fc.up.pt (E.P.); 5 Associate Laboratory i3N, Departamento de Ciência dos Materiais, Faculdade de Ciências e Tecnologia, Universidade NOVA de Lisboa, and CEMOP/UNINOVA, 2829-516 Caparica, Portugal; daniela.gomes@fct.unl.pt (D.N.); emf@fct.unl.pt (E.F.); rm@uninova.pt (R.M.); hma@fct.unl.pt (H.Á.)

**Keywords:** silver nanostars (AgNS), surface-enhanced Raman spectroscopy (SERS), spectral fingerprint, stroke, plasma samples, principal component analysis (PCA), machine learning (ML)

## Abstract

Stroke affects approximately 12 million individuals annually, necessitating swift diagnosis to avert fatal outcomes. Current hospital imaging protocols often delay treatment, underscoring the need for portable diagnostic solutions. We have investigated silver nanostars (AgNS) incubated with human plasma, deposited on a simple aluminum foil substrate, and utilizing Surface-Enhanced Raman Spectroscopy (SERS) combined with machine learning (ML) to provide a proof-of-concept for rapid differentiation of stroke types. These are the seminal steps for the development of low-cost pre-hospital diagnostics at point-of-care, with potential for improving patient outcomes. The proposed SERS assay aims to classify plasma from stroke patients, differentiating hemorrhagic from ischemic stroke. Silver nanostars were incubated with plasma and spiked with glial fibrillary acidic protein (GFAP), a biomarker elevated in hemorrhagic stroke. SERS spectra were analyzed using ML to distinguish between hemorrhagic and ischemic stroke, mimicked by different concentrations of GFAP. Key innovations include optimized AgNS–plasma incubates formation, controlled plasma-to-AgNS ratios, and a low-cost aluminum foil substrate, enabling results within 15 min. Differential analysis revealed stroke-specific protein profiles, while ML improved classification accuracy through ensemble modeling and feature engineering. The integrated ML model achieved rapid and precise stroke predictions within seconds, demonstrating the assay’s potential for immediate clinical decision-making.

## 1. Introduction

Stroke remains a critical global health issue, caused by impaired blood flow to the brain due to either artery blockages (ischemic stroke) or ruptured vessels (hemorrhagic stroke). Blood interruption drastically reduces oxygen and nutrient supply to brain tissue, leading to substantial and often irreversible tissue damage [1,2]. Stroke is the second leading cause of mortality worldwide and a primary contributor to long-term adult disability, affecting around 12 million people each year. Outcomes following a stroke are heavily dependent on the location and extension of brain injury, which affects neurological functions, recovery chances, and quality of life [3,4,5,6]. Ischemic strokes account for nearly 85% of cases and generally result from blood clots or plaque that obstruct blood flow to the brain, while the remaining 15% of strokes are hemorrhagic, caused by bleeding due to ruptured cerebral vessels. Stroke patient outcomes greatly depend on therapy onset since delays in treatment increase risks of disability and mortality [1]. In fact, “time is brain” since every minute following a stroke results in the loss of approximately 1.9 million neurons [7,8,9]. However, ischemic and hemorrhagic stroke present similar clinical symptoms, and advanced imaging techniques (computed tomography (CT) and magnetic resonance imaging (MRI)) are needed for the differential diagnosis [10]. Identification of stroke type is vital for administering correct therapy since ischemic stroke treatments erroneously administered to hemorrhagic stroke patients can lead to severe complications or death. Misdiagnosis not only risks patient health but also results in inefficient use of medical resources, stressing the need for diagnostic tools that offer high sensitivity and specificity [1]. Thus, rapid and pre-hospital stroke diagnosis is urgently needed to improve stroke patient management, shorten treatment onset, and improve final outcomes. Point-of-care testing (PoCT) provides a promising pathway for rapid differential stroke diagnosis, capable of delivering blood sample results within minutes—even on route to the hospital.

Blood plasma, due to its convenient collection and high concentration of analytes, is an optimal biological fluid for Surface-Enhanced Raman Spectroscopy (SERS)-based diagnostics [11,12,13]. SERS has been applied to plasma for cancer biomarker detection [14], such as identifying exosomal biomarkers for early lung and breast cancer diagnosis, and in detecting viral infections, such as hepatitis or influenza [14,15,16]. Plasma is particularly valuable for identifying biomarkers related to stroke due to its role in vascular events and can support the differentiation of ischemic and hemorrhagic strokes [7,17].

One promising biomarker for differentiating between stroke types is Glial Fibrillary Acidic Protein (GFAP). This proteolytic enzyme is primarily expressed in astrocytes and plays key roles in cell–cell communication, namely, astrocyte–neuron interactions and the maintenance of blood–brain barrier (BBB) integrity. In hemorrhagic stroke (HS), the disruption of the BBB leads to astrocyte damage and the subsequent release of GFAP into the bloodstream [7,18]. This release occurs rapidly due to the acute disruption of the BBB, contrasting with the slower and less pronounced release observed in ischemic stroke (IS). This temporal and quantitative difference makes GFAP a valuable biomarker for distinguishing between stroke types, particularly in the early stages of stroke biological events [7,19,20]. There is a strong correlation between GFAP blood levels and hematoma size, with significantly elevated concentrations observed in HS compared to IS during the first 24 h after stroke onset. This correlation has demonstrated good sensitivity and specificity, particularly in cases involving larger hematomas, making GFAP a promising indicator for stroke differentiation in clinical diagnostics [7,21].

To understand the potential of SERS in stroke diagnostics, it is important to first understand the basics of Raman spectroscopy. In this technique, a monochromatic laser is directed at a sample, causing the light to scatter. Most photons undergo elastic (Rayleigh) scattering, but a fraction experiences Raman scattering, where energy shifts due to molecular vibrations, revealing the molecular structure of the sample [22,23,24]. However, traditional Raman spectroscopy has limitations in sensitivity due to the inherently low probability of Raman scattering from individual molecules. SERS overcomes this challenge by amplifying Raman signals from molecules adsorbed to metal nanostructures, at “hot spots” up to 10^12^-fold [22,23,25].

Silver nanostars (AgNS) are a particularly effective nanostructure in SERS applications due to their unique star-shaped morphology, which provides numerous sharp tips and edges, “hotspots”, where the electromagnetic field is significantly enhanced due to localized surface plasmon resonance (LSPR) [26,27,28]. This enhancement allows AgNS to amplify Raman signals to a degree that enables the detection of even trace amounts of biomolecules, which is especially beneficial for point-of-care diagnostic applications where rapid and accurate detection is needed [26,27,29]. Our previous background research on exploring these AgNSs for SERS applications revealed that the highest SERS enhancements were achieved using an office paper substrate with deposited AgNSs. A limit of detection for rhodamine-6G as low as 11.4 ± 0.2 pg, with an analytical enhancement factor of ≈10^7^, was obtained for this specific analyte [30]. This is particularly relevant for stroke diagnostics, where the rapid and sensitive detection of specific plasma biomarkers can provide essential information about the type and severity of stroke. The ability to tailor AgNS surfaces to selectively interact with target molecules further increases the diagnostic utility of this approach. Recent developments in artificial intelligence (AI) and machine learning (ML) complement the use of AgNS in SERS by allowing rapid processing and analysis of the complex data generated, further increasing diagnostic accuracy and making this method suitable for real-time, point-of-care applications [19,20,31].

Principal Component Analysis (PCA) is a widely applied statistical method in SERS, simplifying complex spectral data by identifying patterns, clusters, or outliers. PCA transforms spectral data into principal components that capture the most significant variance in the data, enabling deeper analysis with important applications in the biomedical field [26,32]. Integration of machine learning with SERS further enhances diagnostic accuracy by allowing for the analysis of complex data sets and pattern identification in SERS spectra that might otherwise go undetected [33,34]. This is especially important for more complex biomedical matrixes (e.g., serum and plasma vs. urine), where ML algorithms further contribute to increased sensitivity and specificity of SERS measurements.

**Figure 1:**
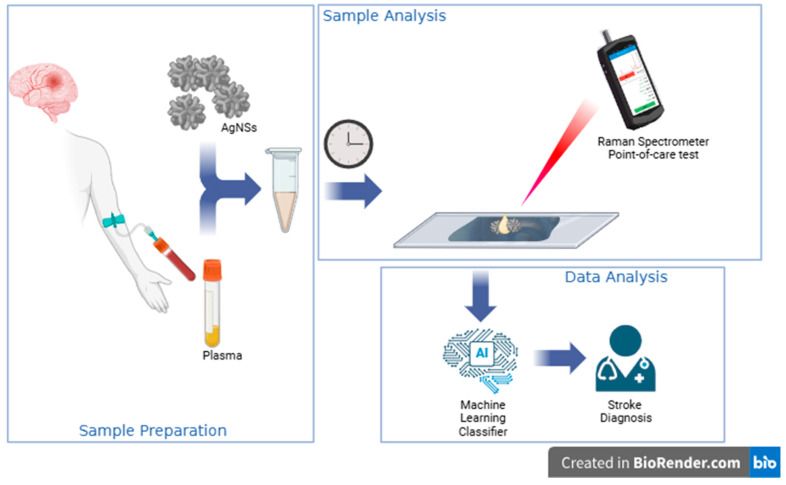
Schematic representation of a stroke diagnostic workflow using surface-enhanced Raman Spectroscopy (SERS) and artificial intelligence (AI). After blood collection from a patient suspected of stroke, centrifugation allows us to obtain plasma (in this work, we have used commercial human plasma spiked with GFAP as a proof-of-concept). Plasma is then mixed with AgNSs and incubated for 15 min before a drop is deposited on an aluminum substrate and analyzed using a Raman spectrometer. We have used a benchtop Raman spectrometer equipped with a microscope, but field use is envisaged with a portable Raman system. The obtained SERS spectra are analyzed using an optimized Machine Learning Classifier. The results can help with the definition of stroke type, and, ultimately, in stroke diagnosis.

Our proposed stroke diagnostic workflow is illustrated in Figure 1. Herein, different experimental SERS conditions were optimized, namely, substrates, concentrations, and time for incubate assembly. For the optimization steps commercial human plasma was used, alone or mixed with recombinant GFAP protein at different concentrations, mimicking ischemic or hemorrhagic stroke patient plasma. Machine Learning Methodology was applied to generate a classifier for distinguishing spectra from different samples. This article presents the first steps for the development of SERS technology as a tool for differential stroke diagnosis, finding future applications on a point-of-care test.

## 2. Materials and Methods

### 2.1. Reagents and Materials

The following reagents were used for the synthesis of silver nanostars (AgNSs): sodium hydroxide solution 98% (Fisher, Waltham, MA, USA) and 50% (*w*/*w*) hydroxylamine solution in water 99.99% (Sigma Aldrich, St. Louis, MO, USA); silver nitrate > 99.99% (Sigma Aldrich, St. Louis, MO, USA) and trisodium citrate dihydrate 99% (Merck, Darmstadt, Germany)

Human plasma obtained from Sigma-Aldrich (St. Louis, MO, USA) was reconstituted in ultrapure water (MilliQ^®^, 18.2 MΩ·cm at 25 °C) and stored in liquid nitrogen aliquots. Before use, plasma aliquots were diluted 1:1 with ultrapure water. Glial Fibrillary Acidic Protein (GFAP), obtained from HyTest, Finland, was diluted to a concentration of 0.1 mg/mL in phosphate-buffered saline (PBS, 1×, pH 7.4, Sigma-Aldrich, St. Louis, MO, USA), prepared with distilled water. Protein concentration was determined by the bicinchoninic acid (BCA) method (based on Smith et al. [35]) using a Sigma-Aldrich kit (St. Louis, MO, USA). For agarose gel electrophoresis (AGE), UltraPure™ Agarose from Bio-Rad and Tris-acetate EDTA (TAE) buffer from Sigma-Aldrich, St. Louis, MO, USA were used. The reagents for the SDS-PAGE included TEMED (>98%) from Tokyo Chemical Industry (TCI, Japan), glycine (>98.5%) from Carlo Erba, ammonium persulfate (10% *w*/*v*), β-mercaptoethanol (>99.0%), sodium dodecyl sulfate (SDS, >98.5%), Brilliant Blue R-250 (Sigma-Aldrich), and glycerol (99.5%) all from Sigma-Aldrich (St. Louis, MO, USA), and bromophenol blue from Panreac AppliChem, Germany. The SDS-PAGE setup utilized 30% (*w*/*v*) acrylamide/bisacrylamide solution and the Mini-PROTEAN system kit from Bio-Rad. The NZYtech Colour protein marker II molecular weight markers set was by NZYtech (Lisbon, Portugal).

### 2.2. Silver Nanostars Synthesis

Silver nanostars were synthesized as described in Garcia-Leis et al. with minor changes [28]. Briefly, a 5.0 mL mixture of equal volumes of 50 mmol·dm^−3^ sodium hydroxide solution and 60 mmol·dm^−3^ hydroxylamine solution was prepared in a beaker. Immediately after, a 1 mmol·dm^−3^ silver nitrate solution was added dropwise (45 mL/min) using a syringe. After 90 s, 0.5 mL of a 1.5% wt. trisodium citrate solution was introduced as a second reducing and capping agent. The reaction was carried out in the dark for 3 h. Following synthesis, the AgNS suspension was centrifuged at 1600× *g* for 12 min, and the resulting pellet was resuspended in ultrapure water before storage in plastic Falcon tubes.

### 2.3. Estimation of Total Protein Molar Concentration in Stock Human Plasma Solution

To prepare plasma@AgNS incubates with controlled molar ratios, we determined the total protein molar concentration of plasma on reconstituted samples to be 55 mg/mL as obtained from the BCA total protein determination. This concentration is close to the lower limit of the estimated total protein concentration in a living human body (60–80 mg/mL). As an approximation, we used the molecular masses and known relative abundances of major plasma proteins, namely, albumin (55%; 66 kDa), globulins (40%; 150 kDa), and fibrinogen (0.05%; 340 kDa), [36] obtaining a weighted plasma proteins molecular weight of 113.3 kDa and a weighted plasma proteins molar concentration of 0.485 M, i.e., 4.85 × 10^8^ nM.

### 2.4. Incubates Assembly

Plasma–AgNS and Plasma–GFAP–AgNS incubates were prepared by simple incubation of AgNSs (0.05 nM) with specific amounts of plasma and/or GFAP to achieve the desired plasma-to-AgNS molar ratios. The incubation was in ultrapure water at 4 °C for 15 min, 3 h, or overnight (ca. 16 h). The amount of plasma to include in the incubates was optimized based on a criterium of a more intense SERS spectrum, presenting a maximum of defined vibrations, and was found to be a plasma-to-AgNS molar ratio of 3.88 × 10^9^, corresponding to a plasma concentration of 0.194 M, i.e., 40% diluted in relation to the stock plasma concentration.

### 2.5. Dynamic Light Scattering Measurements

Dynamic light scattering (DLS) analysis was conducted using a Malvern Zetasizer Nano series (Worcestershire, WR14 1XZ, Malvern, UK). Particle sizes (from 0.3 nm to 10 µm) were determined with single-scattering detection at a 173° scattering angle using a 532 nm diode-pumped, frequency-doubled laser. Measurements were conducted in a temperature-controlled cuvette holder at 25 °C, with samples pre-equilibrated for 5 min. A volume of 800 µL was transferred to a cuvette for DLS (disposable cells). For DLS, each sample was measured in duplicate, with each measurement comprising five acquisitions, while, for zeta potential, each sample was measured in duplicate, with three acquisitions each.

### 2.6. Scanning Electron Microscopy

Scanning electron microscopy (SEM) imaging was employed to confirm the morphology of synthesized AgNSs and Plasma–AgNS incubates. Imaging was performed using a Hitachi Regulus 8220 Scanning Electron Microscope (Mito, Japan) equipped with an Oxford EDS detector. Samples were prepared by drop-casting onto a silicon wafer substrate and allowed to air dry before imaging.

### 2.7. Agarose Gel Electrophoresis

Agarose gel electrophoresis (AGE) was used to assess the size and charge of incubates, demonstrating its successful formation. A horizontal gel electrophoresis system (Bio-Rad Laboratories) operated at 80 V (E = 10 V/cm) was used with 0.3% (*w*/*v*) agarose (UltraPure™ Agarose, Invitrogen) in 0.5× TAE buffer. Incubates were obtained overnight at 4 °C and then centrifuged at 9500× *g* for 10 min at 4 °C. Pellets were resuspended in 13.5 µL of potassium phosphate buffer (5 mM, pH 7.4), with 1.5 µL glycerol added to improve sample density and facilitate well loading.

Electrophoretic mobility (µ) was quantified as the migration distance (ν) divided by the electric field strength (E), with variations (Δµ) calculated relative to the maximum mobility band. Migration distances were measured using the ImageJ with Java 8 software (https://imagej.nih.gov/ij/, accessed on 13 December 2024). We represent our AGE mobilities as variations relative to the maximum mobility band (∆µ).

### 2.8. Hill Fitting to AGE Data

As plasma proteins attach to the surfaces of AgNSs, the mass of the resulting incubate increases, leading to a decrease in electrophoretic mobility and a reduction in surface negative charge. This behavior is observed as slower migration toward the positive electrode. Using ImageJ (https://imagej.nih.gov/ij/, accessed on 13 December 2024), migration distances were calculated for each concentration ratio based on digital images of the electrophoresis gels, and these data were then fitted to a Hill adsorption isotherm (Equation (1)).

(1) $$\Delta \mu =\frac{{\Delta \mu }_{max}{.x}^{n}}{K_{d}^{n}+x^{n}}$$

In this isotherm model, ∆µ denotes the change in electrophoretic mobility compared to the initial AgNS incubate before any plasma protein binding, while K_d_ represents the binding constant (in M), or the concentration of plasma proteins at which half of ∆µ_max_ is achieved. The Hill model incorporates a cooperativity parameter n, which reflects positive cooperativity (n > 1) when the binding of additional molecules is promoted, and negative cooperativity (n < 1) when it is inhibited by previous binding. When n = 1, there is no cooperativity, allowing the system to be characterized by a Langmuir adsorption isotherm.

### 2.9. SDS-PAGE

SDS-PAGE gels were prepared with 12 % (*v*/*v*) acrylamide for the resolving gel and 5% (*w*/*v*) for the stacking gel, enabling protein separation between 12 and 60 kDa. The samples (10 µL) were run alongside low molecular weight (LMW) markers at 150 V for 1.25 h. The gels were stained with 2% (*w*/*v*) Coomassie Blue R-250 and destained until bands were clearly visible. Plasma@AgNS and (Plasma+GFAP)@AgNS incubates were obtained with 15 min, 3 h, or overnight incubation. The control plasma was 10× diluted in relation to the concentration used to incubate with AgNS, and the control (plasma+GFAP) was, at the same concentration, used to incubate with AgNS and 0.1 mg/mL GFAP. After incubation, equal volumes of the sample and sample buffer were combined, centrifuged for 2500× *g* for 10 min at 4 °C, and heated at 100 °C for two minutes, before being applied to the wells. Migration distances from the wells to the samples, which corresponded to the center of the most intense band, were quantified using ImageJ (National Institutes of Health, https://imagej.nih.gov/ij/, accessed on 13 December 2024).

### 2.10. Raman and SERS Measurements

Spectral acquisition for Surface-Enhanced Raman Spectroscopy (SERS) was conducted using a Renishaw inVia™ Qontor^TM^ confocal Raman microscope (Renishaw, Wotton-under-Edge, UK), equipped with a Peltier cooled to −70 °C for the ultra-low noise charge-coupled device (CCD) Centrus 2957T3 detector and a He–Ne laser operating at 17 mW with a wavelength of 633 nm and diffraction grating with 1800 L/mm. The samples were placed on the opaque side of a square piece of aluminum foil (thickness 12 µm, purchased at a local store). The spectroscopic system had a resolution of 0.3 cm⁻^1^, and the laser beam was focused through a 20× objective lens. To minimize random background noise from the detector, three scans of 5 s each were integrated for all measurements. The incident laser intensity was set at 3.2 mV, and duplicate spectra were recorded for each sample. Between SERS sessions, calibration of the spectrograph was performed using the Raman line at 521 cm⁻^1^ from an internal silicon wafer. This calibration procedure verified both the wavenumber scale accuracy and the stability of the Raman signal intensity to mitigate possible fluctuations. To further minimize variability, care was taken to ensure that the aluminum foil substrate remained as flat as possible during measurements, reducing potential laser focus shifts. Three µL of the plasma@AgNS incubates were dropped onto an aluminum foil substrate. Several SERS maps of the samples were acquired, corresponding to 574 spectra measured within a square area of 24 × 24 μm^2^. The initial laser focus was set at the edge of the droplet, where the sample layer was thinner, allowing for sharper focus. After focusing, one SERS map was collected at the edge of the droplet, and a subsequent map was collected in the center of the droplet for each sample. A 20× objective was used for the measurements, with an approximate focal diameter of 38.6 μm and a focal depth of 3.17 μm, which determined the portion of the droplet contributing to the recorded signal. Raw data were digitally captured using the Wire^TM^ 5.6 software for further processing. Vibrational line areas were analyzed using PeakFit v4.12 software, which facilitated baseline correction and Gaussian deconvolution to pinpoint vibrational levels based on the Lorentzian area.

### 2.11. Machine Learning Methodology

**Figure 2:**
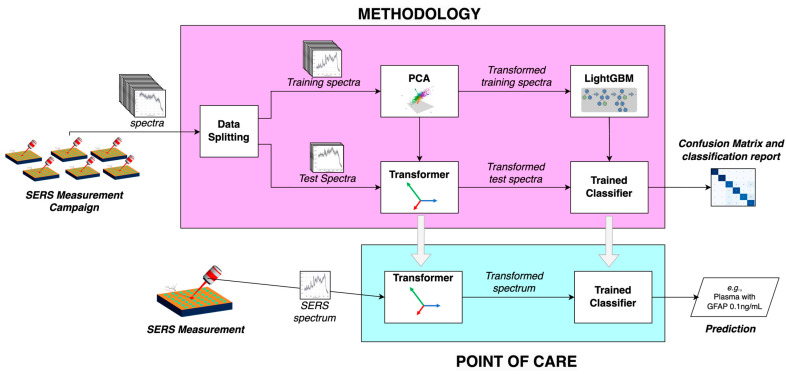
Workflow to classify SERS spectra of incubates of AgNS with plasma.

The Machine Learning methodology is illustrated in Figure 2; in order to train a machine learning model able to classify SERS spectra of plasma with different GFAP concentrations, we undertook the following: (i) data splitting into training and testing sets; (ii) Principal Component Analysis (PCA) and resulting Transformer, for dimensionality reduction; (iii) LightGBM machine learning framework, and resulting trained classifier. The congregation of these components enables us to receive SERS spectra and train a classification model that is capable of predicting a whole. The outcome is a confusion matrix that enables the evaluation of the performance of the developed methodology. For the implementation of a point of care that enables the evaluation of a new spectrum, the Transformer and Trained Classifier are used to provide a prediction of the type of sample.

#### 2.11.1. Data Splitting into Training and Testing Sets

One important step in training a machine learning model is splitting the available data into training and testing data. Training data will be used for training the machine learning model while testing data will enable the performance evaluation of the trained classifier. Shuffling the data before splitting disrupts potential biases or ordering in the dataset, creating a more representative and randomized distribution of samples. This step is particularly crucial for imbalanced or sequential data, ensuring a fair performance evaluation.

#### 2.11.2. Principal Component Analysis

Principal Component Analysis (PCA) is a dimensionality reduction technique that transforms a dataset into a smaller set of uncorrelated variables called principal components while retaining most of the original data’s variance. By identifying the directions (components) of maximum variability in the data, PCA simplifies complex datasets, reduces noise, and mitigates issues like multicollinearity. In machine learning, PCA is often used as a pre-processing step to improve model performance, particularly for high-dimensional data, by reducing computational complexity and focusing on the most informative features of the dataset [37].

#### 2.11.3. Transformer

Once the principal components of the training spectrum are identified by the PCA module, these can be used by a trained or fitted PCA model, a so-called transformer, to transform datasets. It projects datasets onto the computed principal components, providing, in this way, a dimensionality reduction, making the classification of data more efficient.

#### 2.11.4. LightGBM Model

Light Gradient Boosting Machine (LightGBM) is a high-performance machine learning framework designed for gradient boosting that is optimized for speed and efficiency. Boosting algorithms are built using trees: an initial model is created, and each subsequent model is designed to minimize the errors of the previous one. In this way, each new model is a new tree that compensates for the errors made earlier. To construct trees in gradient boosting models, one can employ two primary strategies: level-wise, which grows the tree level by level, and leaf-wise, which selects the leaf that results in the greatest reduction in loss. LightGBM uses the leaf-wise tree growth strategy and supports parallel training, which enhances accuracy by focusing on regions with higher information gain. Another advantage of LightGBM is that it uses a histogram-based algorithm, enabling it to handle large datasets and high-dimensional features effectively. LightGBM supports parallel training and advanced features, like leaf-wise tree growth, which enhances accuracy by focusing on areas with higher information gain. This model excels in classification tasks due to its ability to capture complex relationships in data, manage imbalanced datasets, and handle categorical features natively. Its efficiency and predictive power make it ideal for large-scale classification problems [38].

#### 2.11.5. Trained Classifier

As a result, a trained classification model is obtained. It has learned to recognize patterns in data to assign inputs to specific classes. During the training phase, it analyses labeled training data (identified with the associated class/type) and adjusts its internal parameters to minimize errors in its predictions. Once trained, it can process new, unseen data and predict the most likely class based on the learned patterns. It can perform efficiently and automatically the task of decision-making in identifying the sample type. Testing spectra are used as input to evaluate the performance of the trained model. The evaluation outputs include a confusion matrix and classification report.

The confusion matrix is a valuable tool for assessing classification performance. It summarizes the number of correct and incorrect predictions for each class, categorizing them into true positives, true negatives, false positives, and false negatives. It provides detailed insight into how well the model differentiates the classes [39]. Based on the confusion matrix results, several metrics are computed to evaluate the performance of the model, namely, (i) accuracy, which is the proportion of total correct predictions (both true positives and true negatives) among all predictions made. (ii) Precision, which is the proportion of true positive predictions among all positive predictions made by the model, highlighting the accuracy of positive classifications. This is particularly important when false positives have significant consequences, such as in classifying strokes. (iii) Recall, or sensitivity, which measures the proportion of actual positive cases that were correctly identified, reflecting the model’s ability to detect all relevant instances. (iii) F1-score, which balances these two metrics, providing a harmonic mean that is particularly useful when there is a trade-off between precision and recall [40]. In multi-class classification, where more than two classes are involved, metrics like precision, recall, and F1-score are computed for each class independently, often using a one-vs-rest strategy. In this approach, the model treats each class as the “positive” class while considering all others as the “negative” class. Depending on how the importance of each class is considered in the final evaluation, the metrics are averaged across all classes using macro-average or weighted average; the first considers that all classes are equally important, while the latter weighs each class according to the sample size [41].

## 3. Results and Discussion

Our proposed stroke diagnostic workflow (Figure 1) begins with the collection of blood plasma from a stroke patient in the ambulance. Plasma is mixed with silver nanostars (AgNSs), nanostructures specifically engineered to amplify the Raman signal through surface-enhanced Raman spectroscopy (SERS). When a 633 nm laser (red) is directed at the sample, it generates a unique SERS spectral fingerprint that is highly specific to the chemical and structural properties of plasma molecules adsorbed to the AgNSs, including biomarkers associated with different types of strokes. Here, we have used commercial human plasma spiked with GFAP at different concentrations to investigate the diagnostic potential of the GFAP biomarker in an AgNS–SERS detection system, followed by spectral ML analysis. Three distinct GFAP concentrations and incubation times were selected to simulate varying stroke scenarios. A concentration of 0.1 ng/mL represents the low levels of GFAP typically associated with ischemic stroke within the first 1–3 h after symptom onset. A GFAP concentration of 0.5 ng/mL was used to reflect the intermediate GFAP levels observed in hemorrhagic stroke cases within the first 3 h. Finally, a high concentration of 1 ng/mL was included to mimic the elevated GFAP levels typically measured about 12–18 h after hemorrhagic stroke onset, corresponding to extensive BBB disruption and hematoma. These concentrations align with clinical data demonstrating the progressive increase in GFAP levels with the severity and time after stroke onset [21].

### 3.1. Characterization of Silver Nanostars and AgNSs Incubates with Plasma

**Figure 3:**
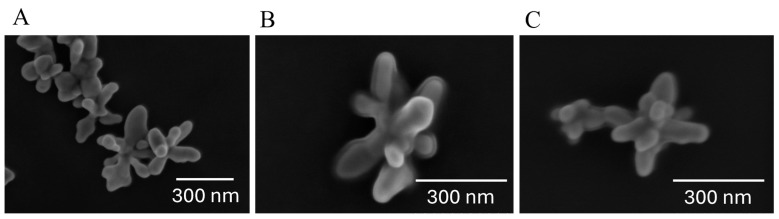
Scanning electron microscopy (SEM) representative micrographs, illustrating the morphological characteristics of native AgNSs, or plasma–AgNS incubates. (**A**) AgNSs in their native form. (**B**) AgNS incubated with plasma for 15 min. (**C**) AgNS incubated with plasma overnight.

The silver nanostars (AgNSs) synthesized for this study were thoroughly characterized using scanning electron microscopy (SEM) (Figure 3). Complementary techniques have provided insights into AgNS–plasma incubation efficiency. Namely, Agarose Gel Electrophoresis (AGE) (Figure S1) and Dynamic Light Scattering (DLS) (Figure S2) were employed to assess the of AgNSs incubates stability with plasma samples spiked with GFAP at varying concentrations and to determine the average diameter of AgNSs alone and their incubates. The SDS-PAGE results of these incubates gave insights into the composition of the protein corona around the AgNSs (Figure S3).

The Scanning Electron Microscopy analysis revealed that AgNSs possess a distinctive morphology characterized by a central core from which multiple elongated spikes radiate outward (Figure 3A). These spikes vary in shape, orientation, and sharpness, leading to a heterogeneous appearance within the same batch. The average tip-to-tip and arm length for the nanostars was 186.8 ± 41.1 nm and 99.1 ± 7.9 nm, respectively, with the observed heterogeneity primarily attributed to variations in spike length and morphology, as seen in the SEM micrograph (Figure 3A).

The formation of incubates between the AgNSs and plasma proteins was observed in the SEM micrographs, where a film of low-contrast material around the AgNSs can be observed (Figure 3B,C). This film is the protein corona, a layer of plasma proteins that adsorbs onto the surface of the nanostars during contact with biological fluids. At low incubation times, an initial protein corona forms, which is kinetically labile and consists mainly of the more abundant proteins loosely bound to the AgNS surface (soft corona). This soft corona slowly transforms into a more densely packed layer of high-affinity proteins (hard corona), existing in blood at lower concentrations. Interestingly, in SEM micrographs, the protein corona was apparent even after a short incubation period of only 15 min (Figure 3B), suggesting that the AgNS surfaces were rapidly and almost completely covered by plasma proteins. This rapid soft corona formation underscores the strong affinity of plasma proteins for the AgNS surface. At a longer incubation time (Figure 3C), a thinner and denser hard corona is visible, as compared to the low contrast of the soft corona, as longer incubation can potentially lead to further stabilization or restructuring of the protein corona around AgNSs.

Agarose Gel Electrophoresis is a powerful tool for the identification of incubates [42]. Protein adsorption at the surface of AgNSs leads to reduced electrophoretic mobility, mainly due to the reduction of the surface charge of AgNSs. Electrophoretic mobility data were plotted versus plasma concentration and fitted to an adsorption isotherm of the Hill type (Figure S1), with a binding constant (K_d_) of 5.0 × 10^9^ M⁻^1^, Δμ_max_ of 7.9 × 10⁻^8^ m^2^V⁻^1^s⁻^1^, and a Hill coefficient (n) of 1.4. The latter indicates that plasma proteins bind to AgNS with positive cooperativity, with one protein favoring the binding of the next (see Materials and Methods for details on the fitting equation). The calculated binding constant (K_d_) is within the range of what has been reported using other techniques, falling anywhere from 10^4^ to 10^11^ M^−1^ [43].

### 3.2. Characterization of AgNSs Incubated with Plasma and GFAP

Dynamic Light Scattering measurements allowed for the determination of the hydrodynamic diameters from AgNSs alone in solution and their respective incubates at different incubation times (Table S1; Figure S2). For AgNS alone, a hydrodynamic diameter of 116 nm with a PDI (polydispersity index) of 0.23 was obtained (Table S1), which is in line with the published results [26]. The formation of robust incubates is highlighted by the red dashed line in the graph of Figure S2, showing that all incubates have hydrodynamic diameters that are ca. 70 nm higher than AgNS, pointing towards a ca. 60% increase in hydrodynamic diameter caused by the plasma corona. This increase is similar for all conditions tested, with ca. 15% larger values observed for overnight incubations of (Plasma+GFAP)@AgNS incubates, likely due to the progressive formation and maturation of a hard protein corona over that longer time period. These findings highlight the dynamic nature of protein corona formation and its stabilization over time while also demonstrating the robustness of the Plasma–GFAP–AgNS conjugation process under different experimental conditions.

The protein composition of the protein corona formed on the surface of the AgNSs in the incubates was analyzed as a function of incubation time using SDS-PAGE. Figure S3 shows the results for the plasma samples (P) incubated with AgNSs for 15 min, 3 h, and overnight. Panel A is the SDS-PAGE for the Plasma@AgNS incubates, while Panel B is the SDS-PAGE for the (Plasma+GFAP)@AgNS incubates. The SDS-PAGE profiles of the incubates reveal proteins from plasma, which adsorb onto the AgNS surface, forming a protein corona. After 15 min of incubation of the Plasma@AgNS incubates (Figure S3A), the protein bands corresponding to major plasma proteins such as albumin (~66 kDa) are prominently visible, indicating their preferential adsorption. As incubation time increases to 3 h and overnight, the protein band from albumin decreases its intensity. This observation hints at a dynamic transition from a “soft” to a “hard” protein corona, with proteins of higher affinity gradually displacing the high-abundance and low-affinity ones, such as albumin, from the silver nanostars surface [44]. For the SDS-PAGE of the plasma spiked with GFAP (Figure S3B), and comparing the profiles for the 15 min, 3 h, and overnight incubation times, an intensity increase in the only band is observed with increasing incubation time, the opposite behavior of what was observed in the absence of GFAP. GFAP itself is not visible on any lanes due, probably, to its low amount. In fact, to remain in line with biologically relevant amounts of plasma and GFAP, only 0.4 μg of pure GFAP was loaded into the (Plasma+GFAP) control lane, an amount close to the lower detection limit for the SDS-PAGE technique, which is 0.3–1 μg of protein per lane [45]. In the control lane, the GFAP band (appearing at ~50 kDa) is probably hidden in the albumin band (~66 kDa band). In the (Plasma+GFAP)@AgNS incubates lanes, no GFAP is visible, probably because the amount of GFAP present in the incubates themselves is much less than what was present in the incubation solution (0.4 μg), this time being well below the lower detection limit for the SDS-PAGE technique. Although further studies are needed to elucidate the complex protein corona dynamics, it is tempting to speculate that GFAP is a low-abundance/high-affinity protein, part of the “hard corona” that, by preferentially adsorbing with high affinity to the surface of AgNS, can also bind to albumin, keeping it on the incubates for the longer incubation times.

These findings highlight the dynamic and time-dependent nature of protein corona formation. Even at short incubation times (15 min), the AgNSs acquire a protein corona capable of supporting biomarker interactions, as seen with GFAP. However, extended incubation times lead to a more complex corona, which may influence the SERS response and biomarker detection sensitivity. These results provide crucial insights into optimizing incubation conditions for point-of-care applications, where rapid corona formation is essential for timely diagnostics.

### 3.3. SERS of AgNSs Incubates with Plasma and GFAP

**Figure 4:**
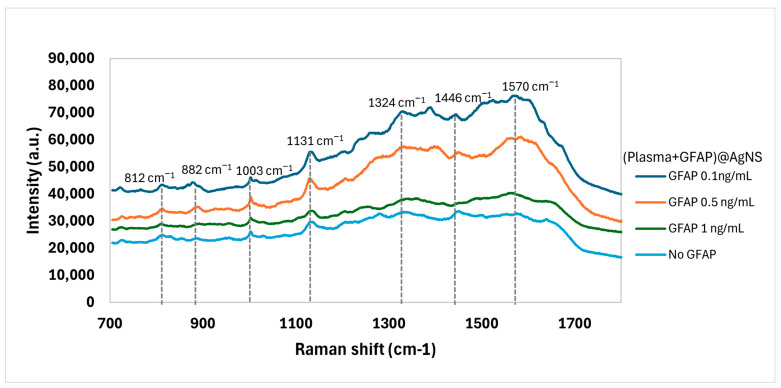
SERS spectra of plasma samples mixed with AgNS and varying concentrations of GFAP (0.1 ng/mL, 0.5 ng/mL, and 1 ng/mL) after 15 min of incubation. The spectrum of plasma without GFAP is shown for comparison. Spectral traces were offset for clarity.

Surface Enhanced Raman Spectroscopy (SERS) spectra were obtained from incubates of AgNSs with plasma and with plasma spiked with GFAP concentrations of 0.1 ng/mL, 0.5 ng/mL, and 1 ng/mL. These concentrations simulate the different stages of a hemorrhagic stroke: within the first hour, after three hours, and on the following day post-stroke [21]. Additionally, different incubation times—15 min, 3 h, and overnight—were evaluated to assess their impact on the SERS spectral profile. The results revealed that, even with the short incubation time of 15 min, with plasma and any of these three GFAP concentrations, it was possible to detect small but notable differences in the SERS spectrum when comparing with plasma alone that was also incubated for 15 min with AgNSs (Figure 4). These differences became more pronounced with longer incubation periods, with overnight incubation providing the most distinct and well-defined spectral features for differentiating between GFAP concentrations, as can be observed in Figure S4. Note that, in both these figures, spectral traces presented were offset for clarity, and the fluorescence contribution to the obtained spectra was negligible. However, at the shortest incubation time (15 min), specific SERS spectral features characteristic of GFAP could be observed, with obvious potential for rapid, in-ambulance diagnostic applications. Machine Learning data analysis was thus applied only to these 15 min-incubation samples.

While visual inspection of the spectra for the 15 min incubation samples with different GFAP concentrations showed some variations in the presence of peaks and the relative intensities, these differences are difficult to quantify based on spectral simulation alone. To detect specific spectral patterns associated with a particular GFAP concentration, machine learning algorithms were employed to detect and classify differences between the three GFAP concentrations at this shortest incubation time of 15 min.

### 3.4. Machine Learning Analysis of AgNSs Incubates with Plasma and GFAP

To evaluate the performance of the proposed methodology, the data set, made up of samples with multiple maps containing various spectra, was divided into training and test sets, as illustrated in Figure 2. In part 3 of the SI (Table S2; Figures S5–S10), average SERS spectra and standard deviation plots are presented for each map obtained for the Plasma@AgNS incubates, GFAP@AgNS incubates, and (Plasma+GFAP)@AgNS incubates, the latter only underwent 15 min of incubation, as those are the conditions that are closer to PoC in-ambulance analysis of patient plasma. A control map was also included in the analysis, namely, of the aluminum foil where samples were deposited for SERS measurement. Due to the limited number of samples, it was impractical to exclude entire samples for testing without compromising the model’s training robustness. Instead, a statistical approach was adopted in which 10% of the spectra from each map were randomly selected for testing, while the remaining 90% were used for training. This ensured that the test data remained representative of the overall dataset while the training set maintained sufficient diversity. To further increase reliability, this training–test split was repeated 1000 times, with the test spectra shuffled in each iteration. After each split, the dataset was randomized to avoid clustering by spectral type. The final evaluation was based on the average performance of all iterations, providing a robust and unbiased assessment of the model’s generalization capabilities. This method made effective use of the available data while maintaining statistical rigor.

**Figure 5:**
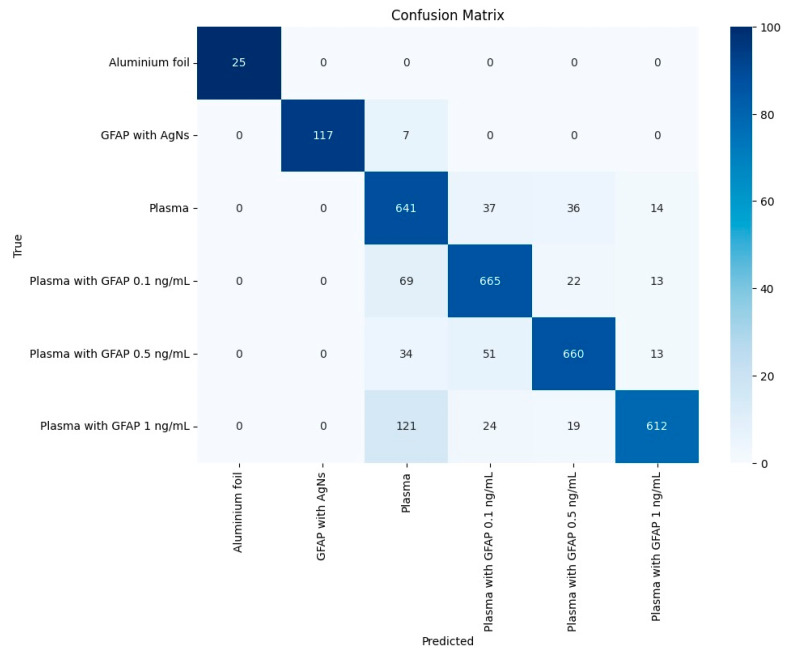
Confusion matrix for one of the testing data sets.

After training the LightGBM Classifier, confusion matrixes are obtained for each of the 1000 splits of training and test data. An example of the resulting confusion matrix for one split is presented in Figure 5. A classification report is built and presented in Table 1, showing results for the proposed metrics, with each value being the average of 1000 obtained values. The results for the testing data demonstrate strong performance of the Classifier, with an overall accuracy of 83%. The model excels in distinguishing the aluminum foil substrate and GFAP with AgNSs, achieving near-perfect precision and recall, which indicates high reliability in identifying this class. Most other classes also show solid results, with GFAP with a plasma concentration of 1 ng/mL achieving a precision of 0.92 and an F1-score of 0.84. However, plasma shows slightly lower precision (0.72) with good recall (0.84), resulting in an F1-score of 0.78. The confusion matrix highlights that misclassifications primarily occur within the GFAP with plasma classes, particularly between the 0.1 ng/mL, 0.5 ng/mL, and 1 ng/mL variants, as well as between plasma and other GFAP categories. These results suggest that, while the model performs well overall, further improvements could be made by addressing these misclassifications.

To evaluate the model’s ability to differentiate the classes, we tested two additional scenarios: one with only samples of GFAP + plasma (GFAP 0.1 ng/mL, GFAP 0.5 ng/mL, and GFAP 1.0 ng/mL), which achieved an accuracy of 87%, and another with GFAP 0.1 ng/mL and GFAP 1.0 ng/mL, which achieved an accuracy of 91%, showing that the model is sensitive to the different concentrations of GFAP used in this study.

To improve the model by reducing false positives and improving the overall classification accuracy, we can perform hyperparameter optimization and data augmentation. Hyperparameters are parameters that control the learning rate and the ability to adjust a model to a specific problem or goal. Their optimization involves systematically searching for the best combination of values. In LightGBM, an example of a hyperparameter is the number of leaves, which controls the complexity of trees and can help reduce false positives. Data augmentation can further improve the model’s robustness by providing additional features. For example, computing statistics like the number of peaks and the signal amplitude can enrich the model’s input, ultimately reducing false positives.

In stroke prediction, precision is critical because false positives can result in misdiagnosis, unnecessary medical interventions, increased costs, and undue stress for patients. It becomes vastly dangerous if a hemorrhagic stroke patient is misdiagnosed as ischemic stroke since its therapy enhances hemorrhage, which can even lead to patient death. A high precision ensures that predictions are trustworthy and minimizes the risks associated with incorrect positive diagnoses. This, in turn, could improve its generalization and reduce errors, which is promising for future applications, including the prediction of stroke nature: Ischemic Stroke (IS) and Intra-Cerebral Hemorrhage (ICH). As the model continues to evolve, it may become a valuable tool in early stroke detection, where distinguishing between various biomarkers and their concentration levels is critical for accurate diagnosis and timely intervention.

## 4. Conclusions

Characterization results of AgNSs and their incubates with plasma and GFAP, using SEM, AGE, DLS, and SDS-PAGE, demonstrate that the synthesized AgNSs possess the structural features and stability required for efficient SERS analysis. The rapid formation of a protein corona upon exposure to human plasma highlights the suitability of these AgNSs as sensitive probes deposited on a low-cost aluminum foil substrate for SERS-based diagnostic assays.

To detect specific spectral patterns associated with a particular GFAP concentration, machine learning algorithms were employed to detect and classify differences between the three GFAP concentrations, at the shortest incubation time of 15 min. This approach holds significant promise for point-of-care diagnostics, as it enables rapid analysis and early-stage discrimination of GFAP levels with minimal incubation. Such rapid diagnostic capability is critical in stroke management, as faster biomarker detection can directly inform treatment strategies, particularly in pre-hospital or emergency settings.

In terms of translating the proof-of-concept system proposed here to actual Point of Care (PoC) use, two main issues must be addressed: (i) preparation of the incubates needs to be simplified, which can be achieved with a plasma separation cartridge to apply to the blood sample and pre-dosed AgNS solutions that can be easily mixed with a pre-set volume of plasma; (ii) SERS spectra must be collected with a portable Raman system, in an optimized setting, and a minimal number of spectra must be established. Once a transformer (fitted PCA) is determined and the classifier is trained, a PoC device can be built and used to rapidly evaluate a SERS spectrum from a patient and classify stroke type. This PoC can be integrated into portable devices for clinical use, enabling real-time analysis and facilitating medical decision-making in emergency scenarios. In addition, the implementation of PoC promotes greater efficiency in diagnosis, reducing the time between analysis and treatment, especially in critical care environments.

## Figures and Tables

**Table 1:** LGBM classification report.

Sample Type	Precision	Recall	F1-Score	Support
Aluminum foil	1.00	0.98	0.99	25
GFAP with AgNS	0.99	0.98	0.98	124
Plasma	0.72	0.84	0.78	728
Plasma with GFAP 0.1 ng/mL	0.82	0.84	0.83	769
Plasma with GFAP 0.5 ng/mL	0.87	0.85	0.86	758
Plasma with GFAP 1 ng/mL	0.92	0.77	0.84	776
Accuracy			0.83	3180
Macro Avg	0.89	0.88	0.88	3180
Weighted Avg	0.84	0.83	0.83	3180

## Data Availability

The data used in the present research are publicly available at the following URL: https://github.com/deisi-r-d/sers-repo (accessed on 1 February 2025).

## Supplementary Materials

The following supporting information can be downloaded at: https://www.mdpi.com/article/10.3390/bios15030136/s1, Figure S1. Agarose Gel Electrophoresis of Plasma@AgNS incubates at varying plasma to AgNS molar ratios.; Table S1. DLS determination of Z-average and standard deviations of hydrodynamic diameters of AgNS alone and Plasma(+GFAP)@AgNS incubates at three different incubation times; Figure S2. Bar graph representation of Table S1; Figure S3. SDS-PAGE of (Plasma)@AgNS and (Plasma+GFAP)@AgNS incubates prepared with increasing incubation times (15 min, 3 h, and overnight).; Figure S4. SERS spectra of plasma samples mixed with AgNS and varying concentrations of GFAP (0.1 ng/mL, 0.5 ng/mL, and 1 ng/mL) after 3 h or overnight incubation.; Figures S5–S10. Plots of averaged SERS spectra and standard deviations for each SERS map.

## References

[1] Hovsepian D., Karceski S. (2013). Stroke, tPA, and Physician Decision-Making. Neurology.

[2] About Stroke, American Stroke Association ASA. https://www.stroke.org/en/about-stroke.

[3] Kowalski R.G., Ledreux A., Violette J.E., Neumann R.T., Ornelas D., Yu X., Griffiths S.G., Lewis S., Nash P., Monte A.A. (2023). Rapid Activation of Neuroinflammation in Stroke: Plasma and Extracellular Vesicles Obtained on a Mobile Stroke Unit. Stroke.

[4] The GBD 2016 Lifetime Risk of Stroke Collaborators (2018). Global, Regional, and Country-Specific Lifetime Risks of Stroke, 1990 and 2016. N. Engl. J. Med..

[5] Lekoubou A., Nguyen C., Kwon M., Nyalundja A.D., Agrawal A. (2023). Post-Stroke Everything. Curr. Neurol. Neurosci. Rep..

[6] Shehjar F., Maktabi B., Rahman Z.A., Bahader G.A., James A.W., Naqvi A., Mahajan R., Shah Z.A. (2023). Stroke: Molecular Mechanisms and Therapies: Update on Recent Developments. Neurochem. Int..

[7] Di Biase L., Bonura A., Pecoraro P.M., Carbone S.P., Di Lazzaro V. (2023). Unlocking the Potential of Stroke Blood Biomarkers: Early Diagnosis, Ischemic vs. Haemorrhagic Differentiation and Haemorrhagic Transformation Risk: A Comprehensive Review. Int. J. Mol. Sci..

[8] Risitano A., Toni D. (2020). Time Is Brain: Timing of Revascularization of Brain Arteries in Stroke. Eur. Heart J. Suppl..

[9] Saver J.L. (2006). Time Is Brain—Quantified. Stroke.

[10] GBD 2019 Stroke Collaborators (2021). Global, Regional, and National Burden of Stroke and Its Risk Factors, 1990–2019: A Systematic Analysis for the Global Burden of Disease Study 2019. Lancet Neurol..

[11] Qin C., Zhao X.-L., Ma X.-T., Zhou L.-Q., Wu L., Shang K., Wang W., Tian D.-S. (2019). Proteomic Profiling of Plasma Biomarkers in Acute Ischemic Stroke Due to Large Vessel Occlusion. J. Transl. Med..

[12] Parachalil D.R., McIntyre J., Byrne H.J. (2020). Potential of Raman Spectroscopy for the Analysis of Plasma/Serum in the Liquid State: Recent Advances. Anal. Bioanal. Chem..

[13] Balogun W.G., Zetterberg H., Blennow K., Karikari T.K. (2023). Plasma Biomarkers for Neurodegenerative Disorders: Ready for Prime Time?. Curr. Opin. Psychiatry.

[14] Beeram R., Vepa K.R., Soma V.R. (2023). Recent Trends in SERS-Based Plasmonic Sensors for Disease Diagnostics, Biomolecules Detection, and Machine Learning Techniques. Biosensors.

[15] Bonifacio A., Dalla Marta S., Spizzo R., Cervo S., Steffan A., Colombatti A., Sergo V. (2014). Surface-Enhanced Raman Spectroscopy of Blood Plasma and Serum Using Ag and Au Nanoparticles: A Systematic Study. Anal. Bioanal. Chem..

[16] Das S.K., Bhattacharya T.S., Ghosh M., Chowdhury J. (2021). Probing Blood Plasma Samples for the Detection of Diabetes Using SERS Aided by PCA and LDA Multivariate Data Analyses. New J. Chem..

[17] Marto J.P., Carvalho A.S., G (2023). Mollet, I.; Mendonça, M.; Salavisa, M.; Meira, B.; Fernandes, M.; Serrazina, F.; Cabral, G.; Ventura, R.; et al. Proteomics to Identify New Blood Biomarkers for Diagnosing Patients With Acute Stroke. J. Am. Heart Assoc..

[18] Foerch C. (2006). Serum Glial Fibrillary Acidic Protein as a Biomarker for Intracerebral Haemorrhage in Patients with Acute Stroke. J. Neurol. Neurosurg. Psychiatry.

[19] Vázquez-Iglesias L., Stanfoca Casagrande G.M., García-Lojo D., Ferro Leal L., Ngo T.A., Pérez-Juste J., Reis R.M., Kant K., Pastoriza-Santos I. (2024). SERS Sensing for Cancer Biomarker: Approaches and Directions. Bioact. Mater..

[20] Xiao R., Zhang X., Rong Z., Xiu B., Yang X., Wang C., Hao W., Zhang Q., Liu Z., Duan C. (2016). Non-Invasive Detection of Hepatocellular Carcinoma Serum Metabolic Profile through Surface-Enhanced Raman Spectroscopy. Nanomed. Nanotechnol. Biol. Med..

[21] Mattila O.S., Ashton N.J., Blennow K., Zetterberg H., Harve-Rytsälä H., Pihlasviita S., Ritvonen J., Sibolt G., Nukarinen T., Curtze S. (2021). Ultra-Early Differential Diagnosis of Acute Cerebral Ischemia and Hemorrhagic Stroke by Measuring the Prehospital Release Rate of GFAP. Clin. Chem..

[22] Long L., Ju W., Yang H.-Y., Li Z. (2022). Dimensional Design for Surface-Enhanced Raman Spectroscopy. ACS Mater. Au.

[23] Stiles P.L., Dieringer J.A., Shah N.C., Van Duyne R.P. (2008). Surface-Enhanced Raman Spectroscopy. Annu. Rev. Anal. Chem..

[24] Solís D.M., Taboada J.M., Obelleiro F., Liz-Marzán L.M., García De Abajo F.J. (2017). Optimization of Nanoparticle-Based SERS Substrates through Large-Scale Realistic Simulations. ACS Photonics.

[25] Araújo A., Caro C., Mendes M.J., Nunes D., Fortunato E., Franco R., Águas H., Martins R. (2014). Highly Efficient Nanoplasmonic SERS on Cardboard Packaging Substrates. Nanotechnology.

[26] De Almeida M.P., Rodrigues C., Novais Â., Grosso F., Leopold N., Peixe L., Franco R., Pereira E. (2023). Silver Nanostar-Based SERS for the Discrimination of Clinically Relevant Acinetobacter Baumannii and Klebsiella Pneumoniae Species and Clones. Biosensors.

[27] De Almeida M.P., Leopold N., Franco R., Pereira E. (2019). Expedite SERS Fingerprinting of Portuguese White Wines Using Plasmonic Silver Nanostars. Front. Chem..

[28] Garcia-Leis A., Garcia-Ramos J.V., Sanchez-Cortes S. (2013). Silver Nanostars with High SERS Performance. J. Phys. Chem. C.

[29] Rodríguez-Lorenzo L., Álvarez-Puebla R.A., De Abajo F.J.G., Liz-Marzán L.M. (2010). Surface Enhanced Raman Scattering Using Star-Shaped Gold Colloidal Nanoparticles. J. Phys. Chem. C.

[30] Oliveira M.J., Quaresma P., Peixoto De Almeida M., Araújo A., Pereira E., Fortunato E., Martins R., Franco R., Águas H. (2017). Office Paper Decorated with Silver Nanostars—An Alternative Cost Effective Platform for Trace Analyte Detection by SERS. Sci. Rep..

[31] Tripathy S., Chavva S., Coté G.L., Mabbott S. (2023). Modular and Handheld Raman Systems for SERS-Based Point-of-Care Diagnostics. Curr. Opin. Biomed. Eng..

[32] Moisoiu T., Iancu S.D., Burghelea D., Dragomir M.P., Iacob G., Stefancu A., Cozan R.G., Antal O., Bálint Z., Muntean V. (2022). SERS Liquid Biopsy Profiling of Serum for the Diagnosis of Kidney Cancer. Biomedicines.

[33] Cui L., Fan Z., Yang Y., Liu R., Wang D., Feng Y., Lu J., Fan Y. (2022). Deep Learning in Ischemic Stroke Imaging Analysis: A Comprehensive Review. BioMed Res. Int..

[34] Iancu S.D., Cozan R.G., Stefancu A., David M., Moisoiu T., Moroz-Dubenco C., Bajcsi A., Chira C., Andreica A., Leopold L.F. (2022). SERS Liquid Biopsy in Breast Cancer. What Can We Learn from SERS on Serum and Urine?. Spectrochim. Acta. A Mol. Biomol. Spectrosc..

[35] Smith P.K., Krohn R.I., Hermanson G.T., Mallia A.K., Gartner F.H., Provenzano M.D., Fujimoto E.K., Goeke N.M., Olson B.J., Klenk D.C. (1985). Measurement of Protein Using Bicinchoninic Acid. Anal. Biochem..

[36] Leeman M., Choi J., Hansson S., Storm M.U., Nilsson L. (2018). Proteins and Antibodies in Serum, Plasma, and Whole Blood—Size Characterization Using Asymmetrical Flow Field-Flow Fractionation (AF4). Anal. Bioanal. Chem..

[37] Jolliffe I.T., Cadima J. (2016). Principal Component Analysis: A Review and Recent Developments. Philos. Trans. R. Soc. Math. Phys. Eng. Sci..

[38] Ke G., Meng Q., Finley T., Wang T., Chen W., Ma W., Ye Q., Liu T.-Y. (2017). LightGBM: A Highly Efficient Gradient Boosting Decision Tree. Proceedings of the 31st International Conference on Neural Information Processing Systems.

[39] Heydarian M., Doyle T.E., Samavi R. (2022). MLCM: Multi-Label Confusion Matrix. IEEE Access.

[40] Erickson B.J., Kitamura F. (2021). Magician’s Corner: 9. Performance Metrics for Machine Learning Models. Radiol. Artif. Intell..

[41] Pedregosa F., Varoquaux G., Gramfort A., Michel V., Thirion B., Grisel O., Blondel M., Prettenhofer P., Weiss R., Dubourg V. (2011). Scikit-Learn: Machine Learning in Python. J. Mach. Learn. Res..

[42] Franco R., Pereira E., Kretsinger R.H., Uversky V.N., Permyakov E.A. (2013). Gold Nanoparticles and Proteins, Interaction. Encyclopedia of Metalloproteins.

[43] Boulos S.P., Davis T.A., Yang J.A., Lohse S.E., Alkilany A.M., Holland L.A., Murphy C.J. (2013). Nanoparticle–Protein Interactions: A Thermodynamic and Kinetic Study of the Adsorption of Bovine Serum Albumin to Gold Nanoparticle Surfaces. Langmuir.

[44] Lynch I., Dawson K.A. (2008). Protein-Nanoparticle Interactions. Nano Today.

[45] Gallagher S., Sasse J. (2001). Protein Analysis by SDS-PAGE and Detection by Coomassie Blue or Silver Staining. Curr. Protoc. Pharmacol..

