# Supplementary material for: Towards Rapid and Low-Cost Stroke Detection Using SERS and Machine Learning
Source: Biosensors (Basel). 2025 Feb 22;15(3):136. doi: 10.3390/bios15030136 (PMC11940671; doi:10.3390/bios15030136)
Supplement: Supplementary file 1 [file biosensors-15-00136-s001.zip › biosensors-3429215-supplementary.pdf]

biosensors

MDPI

Supplementary Information

Article

Towards Rapid and Low-Cost Stroke Detection Using SERS and Machine Learning

Cristina Freitas [1,2], João Eleutério [3], Gabriela Soares [3], Maria Enea [4], Daniela Nunes [5], Elvira Fortunato [5], Rodrigo Martins [5], Hugo Águas [5], Eulália Pereira [4], Helena L. A. Vieira [1,2], Lúcio Studer Ferreira [3,*] and Ricardo Franco [1,2,*]

[1] Associate Laboratory i4HB—Institute for Health and Bioeconomy, Faculdade de Ciências e Tecnologia, Universidade NOVA de Lisboa, 2819-516 Caparica, Portugal; cmt.freitas@campus.fct.unl.pt (C.F.); hl.vieira@fct.unl.pt (H.L.A.V.)

[2] UCIBIO—Applied Molecular Biosciences Unit, Departamento de Química, Faculdade de Ciências e Tecnologia, Universidade NOVA de Lisboa, 2819-516 Caparica, Portugal

[3] COPELABS — Departamento de Engenharia Informática e Sistemas de Informação, Universidade Lusófona, Centro Universitário de Lisboa, 1749-024 Lisboa, Portugal; a22308295@alunos.ulht.pt (J.E.); p7333@ulusofona.pt (G.S.); lucio.studer@ulusofona.pt (L.F.)

[4] LAQV/REQUIMTE—Laboratório Associado para a Química Verde/Rede de Química e Tecnologia, Departamento de Química e Bioquímica, Faculdade de Ciências, Universidade do Porto, 4169-007 Porto, Portugal; menea@fc.up.pt (M.E.); eulalia.pereira@fc.up.pt (E.P.)

[5] Associate Laboratory i3N, Departamento de Ciência dos Materiais, Faculdade de Ciências e Tecnologia, Universidade NOVA de Lisboa, and CEMOP/UNINOVA, 2829-516 Caparica, Portugal; daniela.gomes@fct.unl.pt (D.N.); emf@fct.unl.pt (E.F.); rm@uninova.pt (R.M.); hma@fct.unl.pt (H.Á.)

* Correspondence: lucio.studer@ulusofona.pt (L.S.F.); ricardo.franco@fct.unl.pt (R.F.)

1. Characterization of Plasma(+GFAP)@AgNS incubates

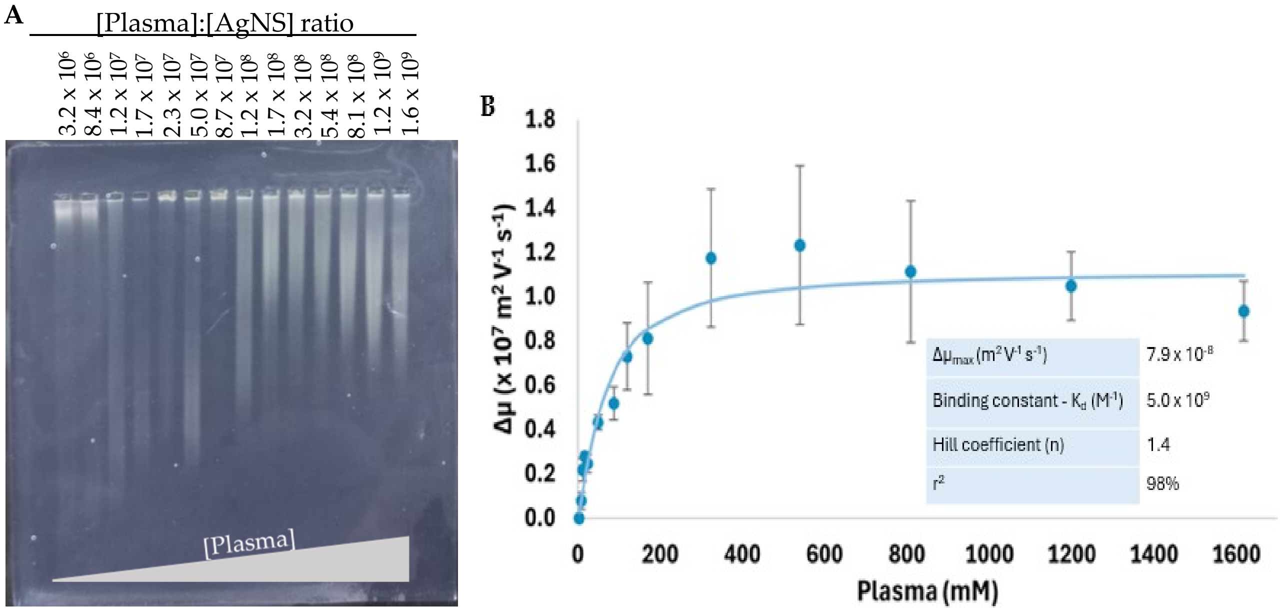

Figure S1. Agarose Gel Electrophoresis of Plasma@AgNS incubates at varying plasma to AgNS molar ratios. (a) Representative AGE of Plasma@AgNS incubates at increasing plasma concentrations. (b) Plot of electrophoretic mobility (Δμ) *vs.* plasma concentration. The line in blue corresponds fitting to a Hill-type equation. Fitting parameters are $\Delta\mu_{max} = 7.9 \times 10^{-8}$ $m^2V^{-1}s^{-1}$, a binding constant (K_d) of 5.0×10^9 M^{-1}, a Hill coefficient (n) of 1.4, and an R^2 value for the fitting of 98%. Error bars represent standard deviations calculated from three independent AGE experiments.

.

Table S1. DLS determination of Z-average and standard deviation (StD) of hydrodynamic diameters of AgNS alone and Plasma(+GFAP)@AgNS incubates at three different incubation times.

		**15 min incubation**			**3 h incubation**			**Overnight incubation**		
	**Sample Type**	**Z-average (nm)**	**StD**	**PDI**	**Z-average (nm)**	**StD**	**PDI**	**Z-average (nm)**	**StD**	**PDI**
	AgNS	116	0.79	0.23						
	Plasma@AgNS	180	5.40	0.13	198	2.73	0.14	180	1.76	0.14
(Plasma+GFAP)@AgNS	GFAP 0.1 ng/mL	193	3.66	0.16	198	1.77	0.13	217	1.62	0.10
	GFAP 0.5 ng/mL	178	1.96	0.14	190	2.11	0.12	214	1.48	0.08
	GFAP 1 ng/mL	191	4.03	0.14	185	1.70	0.12	201	4.20	0.10

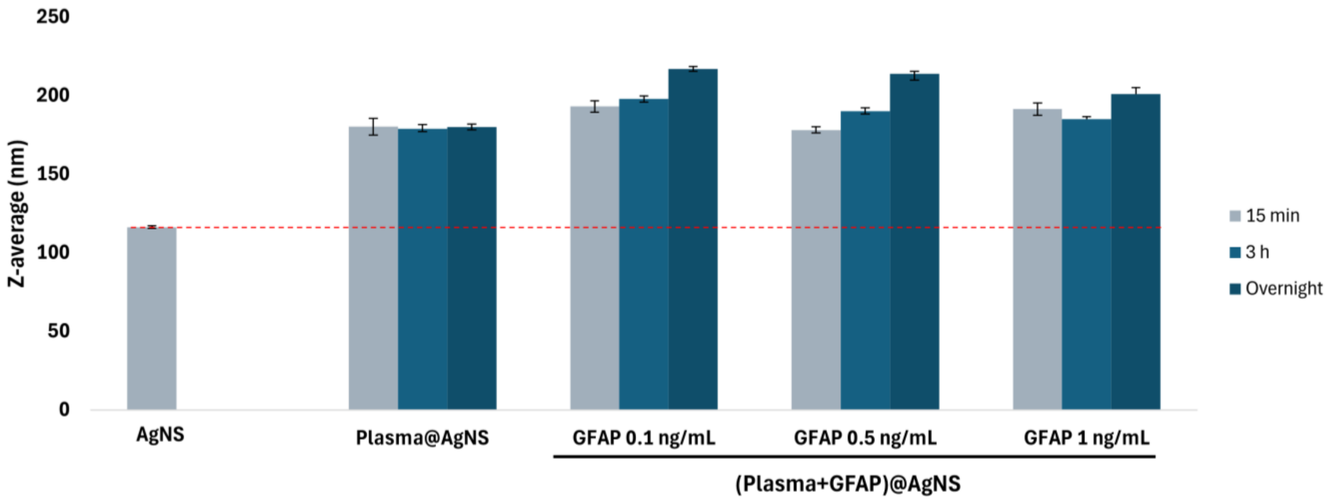

Figure S2. Bar graph representation of Table S1, corresponding to DLS measurements of Z-average (nm) for plasma@AgNS and (plasma+GFAP)@AgNS, at three different GFAP concentrations. Results are shown for incubation times of AgNS and the respective plasma sample, of 15 min, 3 h, and overnight. Error bars represent standard deviations from two independent measurements. A red dashed line was plotted the hydrodynamic diameter for AgNS alone, highlighting the formation of a protein corona on the incubates.

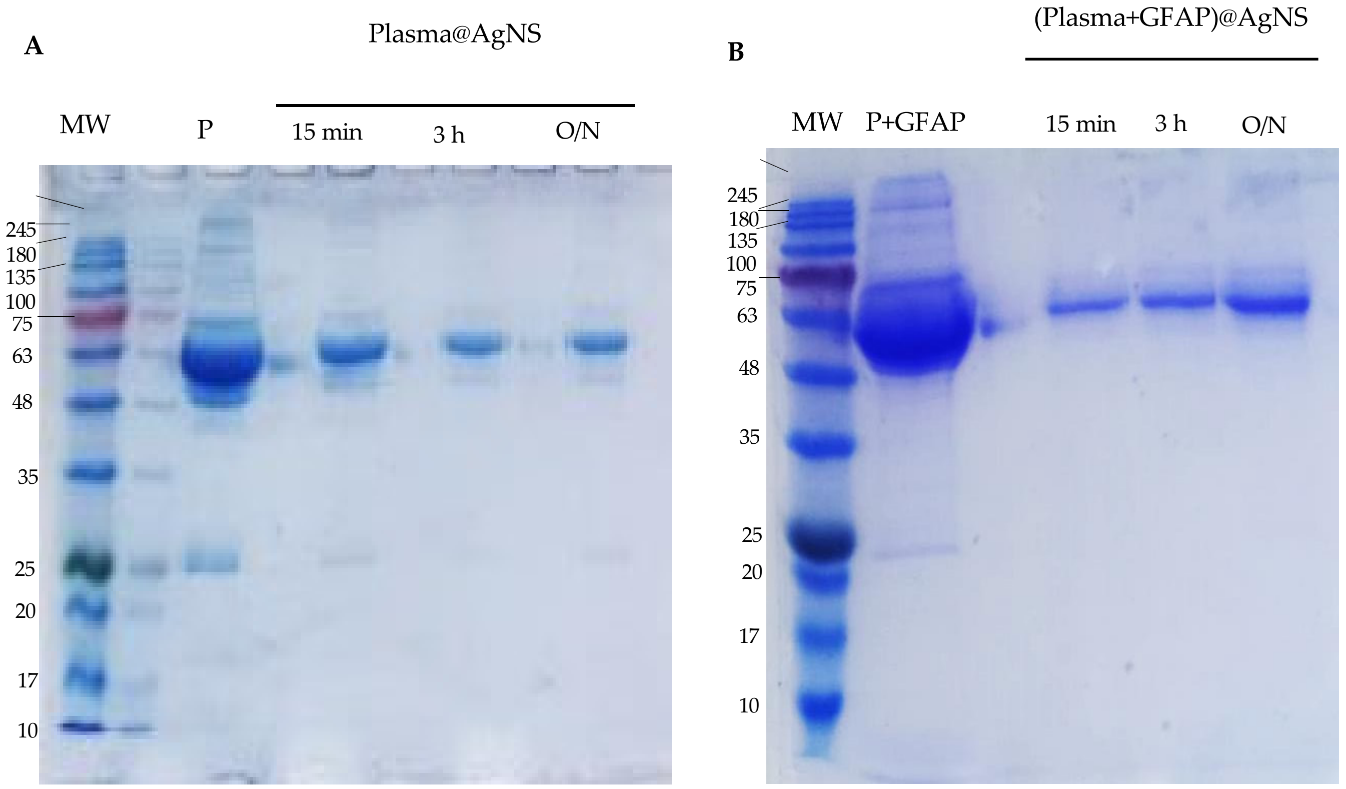

Figure S3. SDS-PAGE of incubates prepared with increasing incubation times (15 min, 3h, and overnight). **A**. Plasma (P) incubates with AgNS. **B**. Plasma samples with GFAP (P+GFAP) incuabted with AgNS. Molecular weight markers (MW), ranging from 245 kDa to 10 kDa, are shown on the far-left lanes, for reference.

2. SERS spectra of Plasma:GFAP@AgNS incubates for 3h and overnight incubations

A

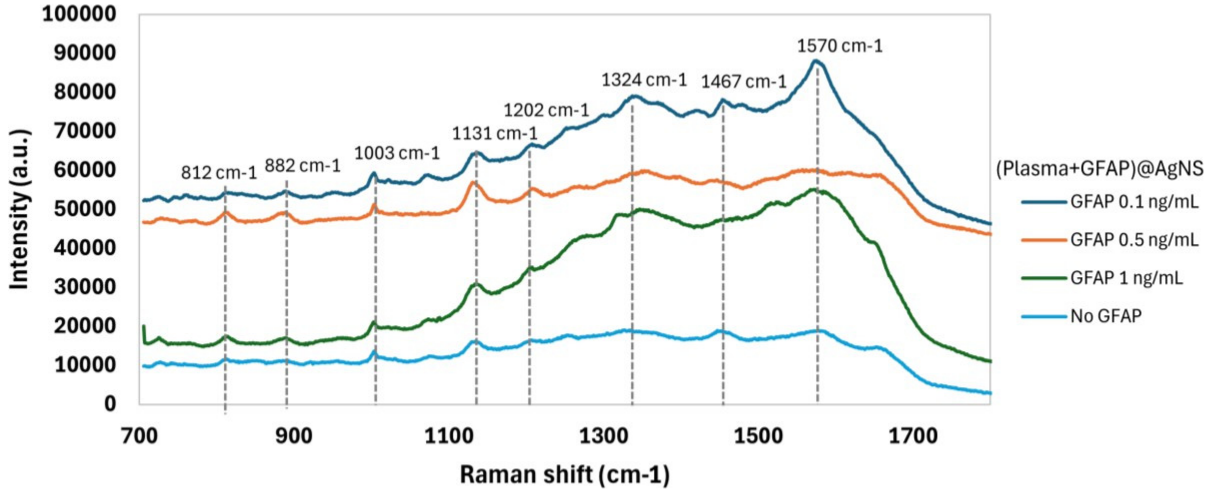

B

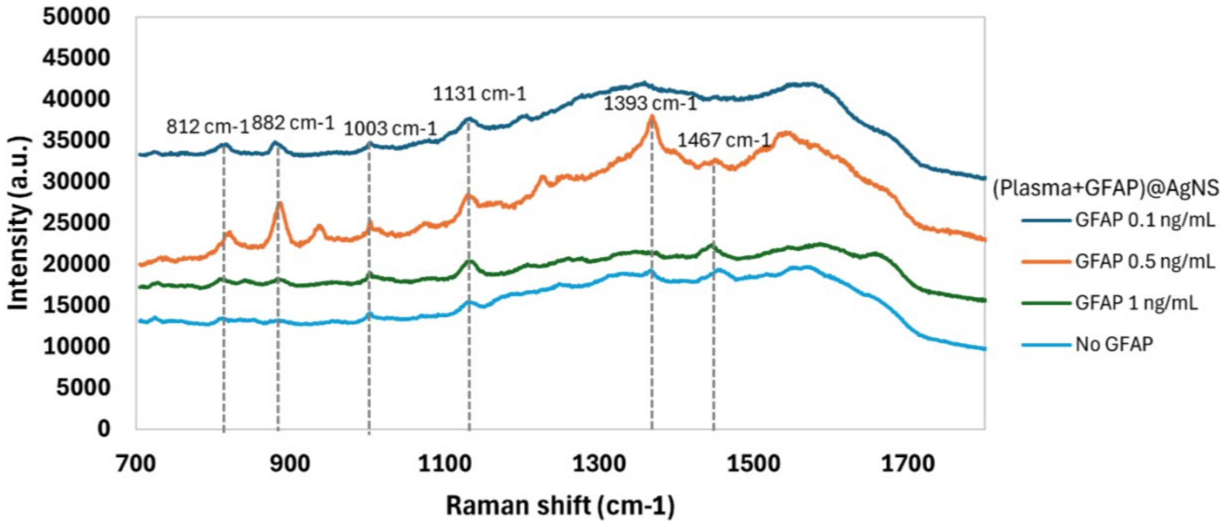

Figure S4. SERS spectra of plasma samples incubated with AgNS and varying concentration of GFAP (0.1 ng/mL, 0.5 ng/mL, and 1 ng/mL) after (A) 3 h incubation and (B) overnight incubation. Spectra of plasma without GFAP are shown for comparison. Spectral traces were offset for clarity.

3. Average SERS spectra and standard deviation for maps of Plasma/GFAP@AgNS incubates with 15 minutes incubation

Description of SERS samples is presented in Table S2. The number of maps per sample type is of the same order of magnitude (around 15), as well as for the number of measured spectra (7500) and their wavenumber range. Regarding the intensity, its variation is large, as expected for the technique.

Table S2. Characterization of SERS samples used in the ML analysis.

Sample Type	Maps	Spectra	Wavenumber Range (cm^{-1})	Intensity Range
Aluminum (sample substrate)	1	256	705.21 - 1820.48	0.00 - 84.17
GFAP@AgNS	2	1250	705.36 - 1820.62	16.40 - 3683.96
Plasma@AgNS	14	7363	705.18 - 1820.58	25.31 - 11038.22
(Plasma + GFAP 0.1ng/mL)@AgNS	17	7770	595.31 - 1820.62	-4.84 - 18738.47
(Plasma + GFAP 0.5ng/mL)@AgNS	14	7655	705.18 - 1820.62	136.57 - 35077.31
(Plasma + GFAP 1ng/mL)@AgNS	15	7843	705.18 - 1820.62	22.68 - 36041.11

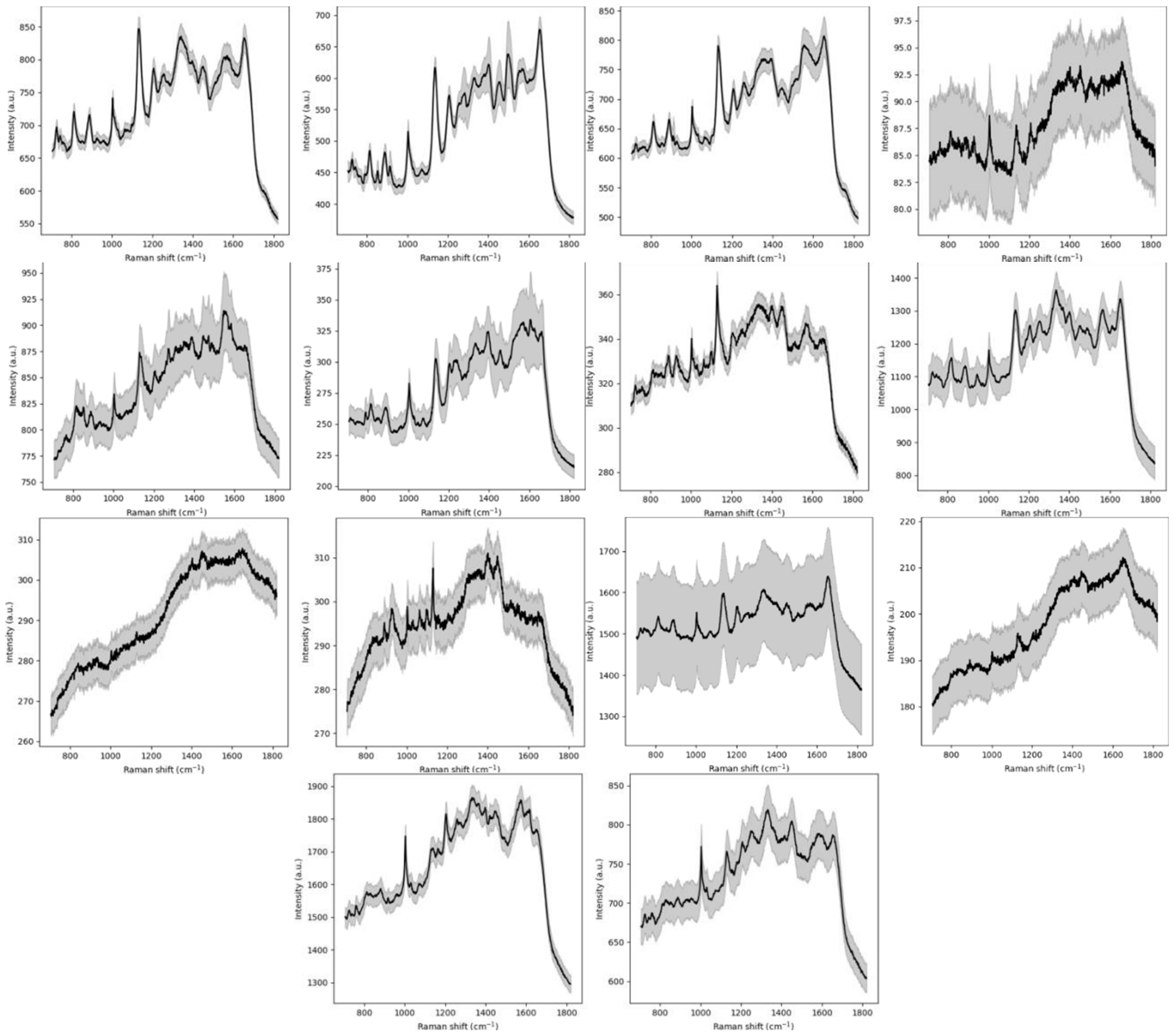

Figure S5. Plots of averaged SERS spectra and standard deviations for each map of Plasma@AgNS incubates samples.

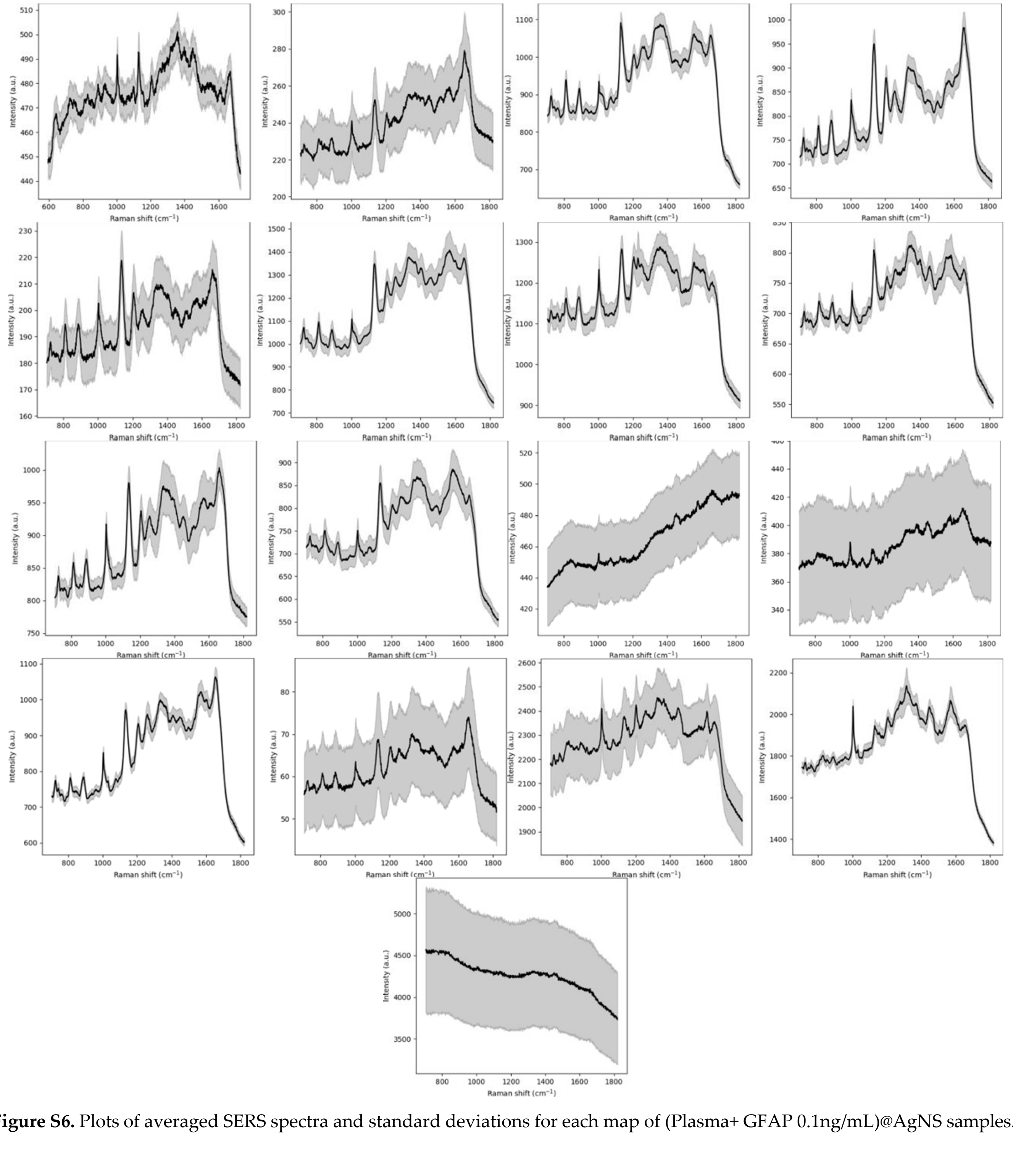

Figure S6. Plots of averaged SERS spectra and standard deviations for each map of (Plasma+ GFAP 0.1ng/mL)@AgNS samples.

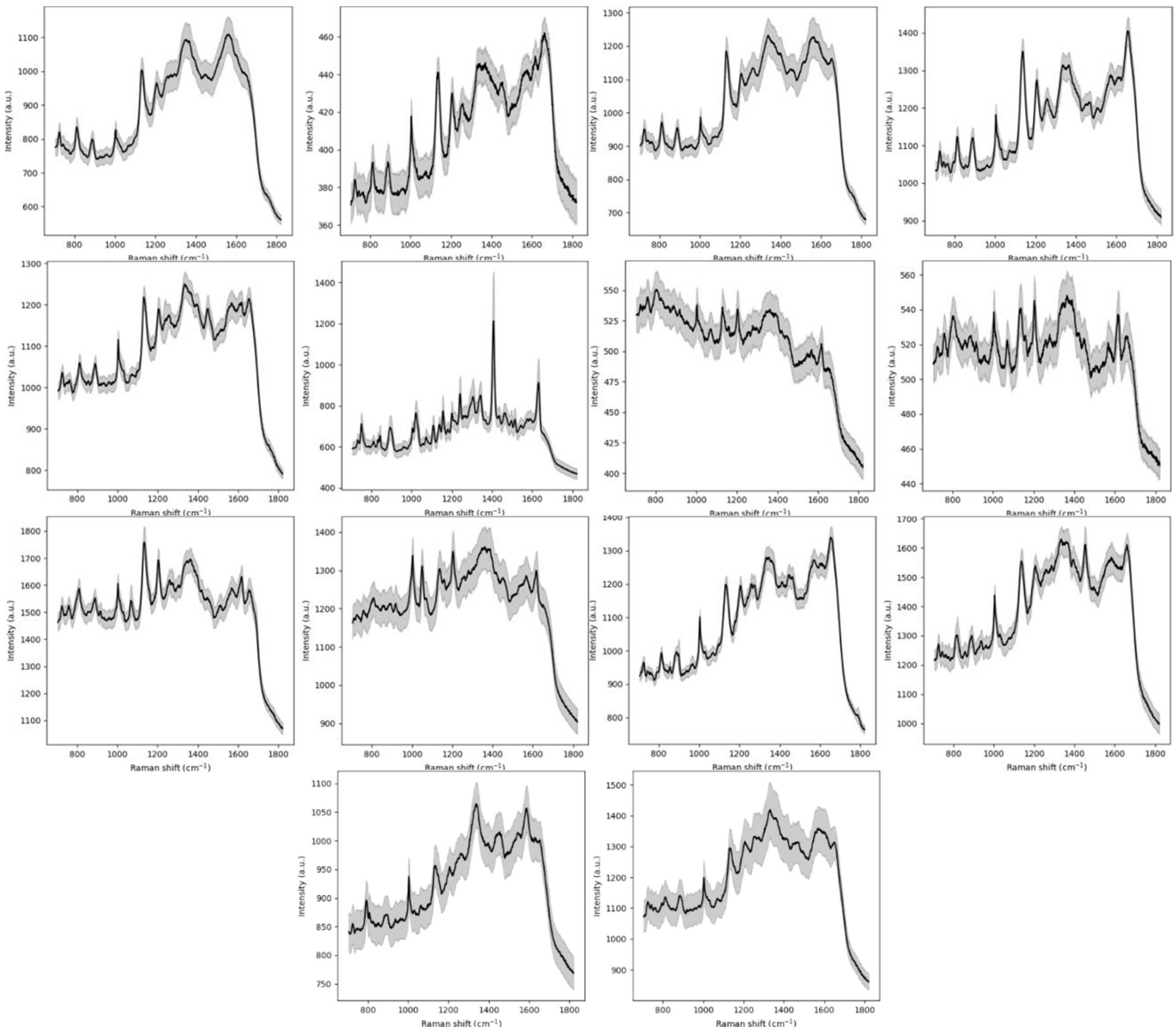

Figure S7. Plots of averaged SERS spectra and standard deviations for each map of (Plasma+ GFAP 0.5ng/mL)@AgNS samples.

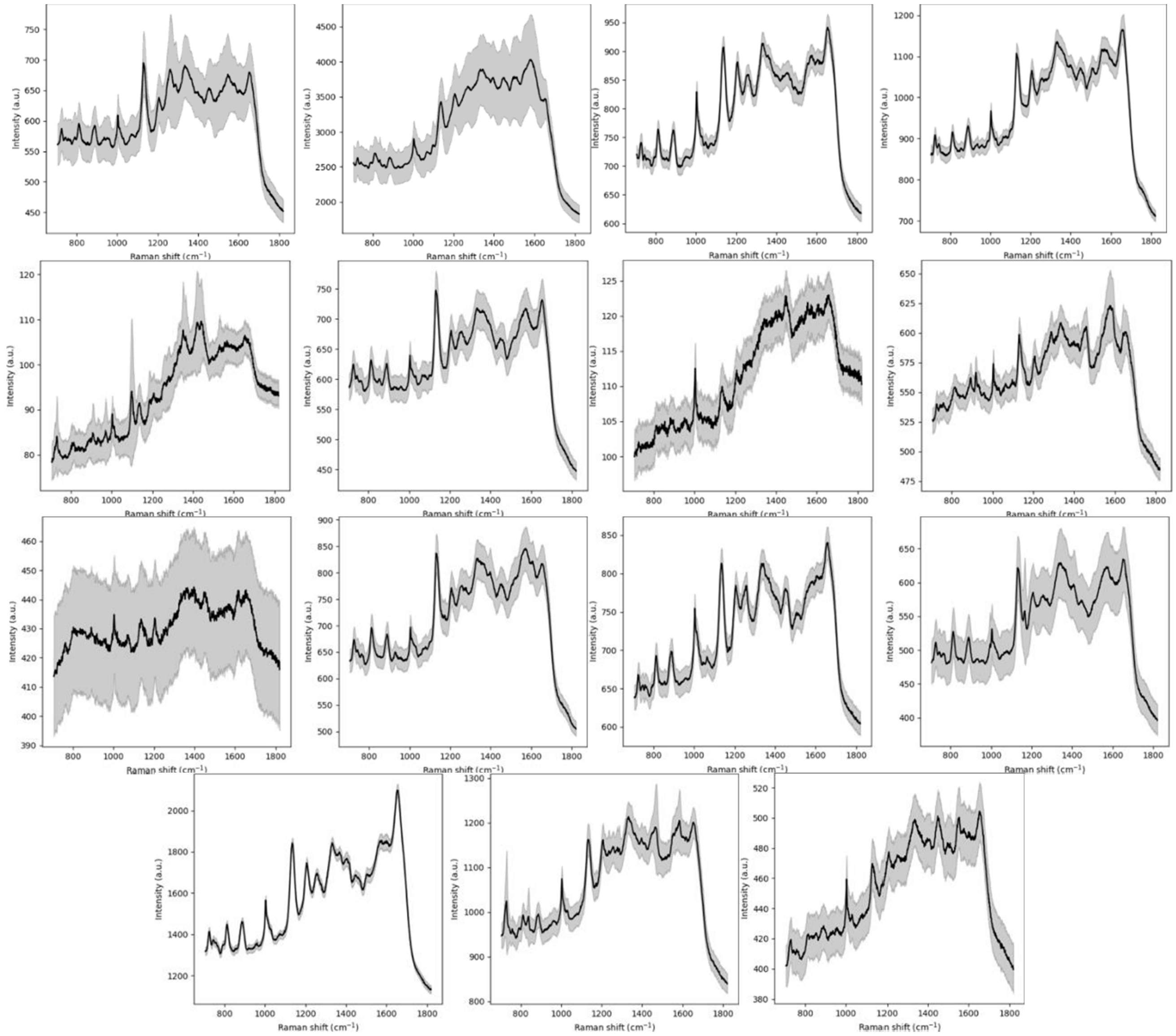

Figure S8. Plots of averaged SERS spectra and standard deviations for each map of (Plasma+ GFAP 1ng/mL)@AgNS samples.

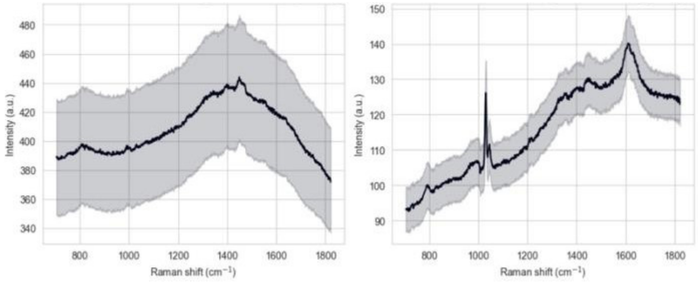

Figure S9. Plot of averaged SERS spectra and standard deviation for the two maps obtained for GFAP@AgNS samples.

.

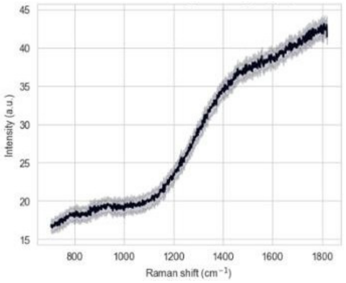

Figure S10. Plot of averaged SERS spectra and standard deviation for a map of the substrate for sample deposition (Aluminum foil).
